# Quantitative Brain Positron Emission Tomography in Female 5XFAD Alzheimer Mice: Pathological Features and Sex-Specific Alterations

**DOI:** 10.3389/fmed.2021.745064

**Published:** 2021-11-26

**Authors:** Caroline Bouter, Caroline Irwin, Timon N. Franke, Nicola Beindorff, Yvonne Bouter

**Affiliations:** ^1^Department of Nuclear Medicine, University Medical Center Göttingen (UMG), Georg-August-University, Göttingen, Germany; ^2^Department of Psychiatry and Psychotherapy, University Medical Center Göttingen (UMG), Georg-August-University, Göttingen, Germany; ^3^Berlin Experimental Radionuclide Imaging Center (BERIC), Charité-Universitätsmedizin Berlin, Berlin, Germany

**Keywords:** 5XFAD Alzheimer model, Alzheimer's disease, positron - emission tomography, ^18^F-Flourdesoxyglucose-PET, ^18^F-Florbetaben-PET

## Abstract

Successful back-translating clinical biomarkers and molecular imaging methods of Alzheimer's disease (AD), including positron emission tomography (PET), are very valuable for the evaluation of new therapeutic strategies and increase the quality of preclinical studies. ^18^F-Fluorodeoxyglucose (FDG)–PET and ^18^F-Florbetaben–PET are clinically established biomarkers capturing two key pathological features of AD. However, the suitability of ^18^F-FDG– and amyloid–PET in the widely used 5XFAD mouse model of AD is still unclear. Furthermore, only data on male 5XFAD mice have been published so far, whereas studies in female mice and possible sex differences in ^18^F-FDG and ^18^F-Florbetaben uptake are missing. The aim of this study was to evaluate the suitability of ^18^F-FDG– and ^18^F-Florbetaben–PET in 7-month-old female 5XFAD and to assess possible sex differences between male and female 5XFAD mice. We could demonstrate that female 5XFAD mice showed a significant reduction in brain glucose metabolism and increased cerebral amyloid deposition compared with wild type animals, in accordance with the pathology seen in AD patients. Furthermore, we showed for the first time that the hypometabolism in 5XFAD mice is gender-dependent and more pronounced in female mice. Therefore, these results support the feasibility of small animal PET imaging with ^18^F-FDG- and ^18^F-Florbetaben in 5XFAD mice in both, male and female animals. Moreover, our findings highlight the need to account for sex differences in studies working with 5XFAD mice.

## Introduction

Alzheimer's disease (AD), the most common form of dementia, affects more than 40 million people worldwide and is characterized by the decline of cognitive abilities and progressive memory loss (1). To date, there is still no cure available, and the current pharmacotherapeutic strategies do not target the underlying molecular processes of the disease (2, 3).

To establish new treatments for AD, both preclinical models of the disease as well as reliable *in vivo* biomarkers are essential. Clinical biomarkers include measurement of Abeta42 and (phosphorylated) tau in cerebrospinal fluid, and also MRI and molecular imaging with positron emission tomography (PET). PET is a functional, non-invasive imaging technique that allows *in vivo* monitoring of pathological and physiological processes (4). ^18^F-Fluorodeoxyglucose (FDG)–PET and ^18^F-Florbetaben–PET are clinically established biomarkers for the diagnosis of AD capturing two key pathological features of the disease (5, 6). FDG–PET can visualize cerebral glucose metabolism that reflects regional neuronal and synaptic activity and can therefore be used to assess synaptic dysfunctions and neuron loss in the brain (7). ^18^F-Florbetaben–PET detects cerebral amyloid load (8). Successful back-translating clinical biomarkers and molecular imaging methods of AD, including PET, is very valuable for the evaluation of new therapeutic strategies and to increase the quality of preclinical studies. However, while PET is an established tool in the assessment of AD patients, its role in preclinical studies using AD mouse models remains unclear.

Several mouse models of AD are available so far showing different pathological hallmarks. Thereby AD models that express mutations in the amyloid precursor protein (APP) and/or presenilin (PS) genes have been widely used to study AD pathology, especially with regard to the Abeta pathology. A well-established and commonly used mouse model is the 5XFAD model, as these mice carry five APP and PS1 mutations that lead to an age-dependent aggressive amyloid plaque pathology, neuronal dysfunction, and neuron loss as well as behavioral deficits (9, 10).

A recent study of our group showed that both ^18^F-FDG- and ^18^F-Florbetaben-PET are suitable tools for the assessment of AD pathologies in male 5XFAD mice *in vivo*, postulating its broader use in longitudinal preclinical therapy studies (11). However, only data on male 5XFAD mice have been published so far whereas possible sex differences in ^18^F-FDG and ^18^F-Florbetaben uptake have not been studied (12–14).

The aim of this study was to evaluate the suitability of ^18^F-FDG– and ^18^F-Florbetaben–PET in female 5XFAD mice and to assess possible sex differences.

## Materials and Methods

### 5XFAD Transgenic Mice

5XFAD mice overexpress the 695 amino acid isoform of the human APP carrying the Florida (I716V), London (V717I), and Swedish (K670N/M671L) mutations under the control of the neuron-specific murine Thy1-promoter. In addition, human presenilin-1 (PS1), which carries the L286V and M146L mutations, is expressed under the control of the Thy-1 promoter (9). 5XFAD mice used in this study were kept on a C57Bl/6J genetic background (15) (Jackson Laboratories, Bar Harbor, ME, USA) and wild type (WT) littermates served as age-matched control animals.

All animals were handled according to the German guidelines for animal care and approved by the local authorities (Niedersächsisches Landesamt für Verbraucherschutz und Lebensmittelsicherheit, Röverskamp 5, 26203 Oldenburg, Germany and Landesamt für Gesundheit und Soziales LAGeSo Darwinstr. 15, 10589 Berlin, Germany).

### ^18^F-FDG-PET/MRI

Seven-month-old female (*n* = 6) and male (*n* = 4) 5XFAD, and female (*n* = 6) C57Bl/6J WT mice were scanned with ^18^F-FDG-PET/MRI (*n* = 4–6 per group) using a small animal 1 Tesla nanoScan PET/MRI (mouse whole body volume coil, diameter 35 mm, Mediso, Hungary) as previously described (11, 16). Mice fasted overnight, and blood glucose levels were measured before tracer injection. Mean activity of 16.2 MBq ^18^F-FDG (11.5–19.5 MBq) was injected into a tail vein with a maximum volume of 200 μl. The uptake period was 45 min in which mice were awake and kept warm. Afterward, mice were anesthetized with 1–2% isoflurane supplemented with oxygen (0.5 l/min, cp-pharma, Burgdorf, Germany). PET acquisition time was 20 min. Scans were performed in a single mouse model. Mice were kept on a heated bed (37°C) during the scans and the respiratory rate was measured. MRI-based attenuation correction was conducted with the material map (matrix 144 × 144 × 163 with a voxel size of 0.5 × 0.5 × 0.6 mm^3^, repetition time: 15 ms, echo time: 2.032 ms, and a flip angle of 25°), and the PET images were reconstructed using the following parameters: matrix 136 × 131 × 315, voxel size 0.23 × 0.3 × 0.3 mm^3^.

### ^18^F-Florbetaben–PET/MRI

Amyloid imaging was performed with the commercially available tracer ^18^F-Florbetaben (NeuraCeq, Life Molecular Imaging, Berlin, Germany) as described previously (11). ^18^F-Florbetaben-PET/MRI was performed on 7-month-old 5XFAD and C57Bl/6J wild type mice (*n* = 4–6 per group). ^18^F-Florbetaben (9.1–21.8 MBq; mean 15.6 MBq) was injected into a tail vein with a maximum volume of 200 μl. PET acquisition time was 30 min starting after an uptake period of 40 min. Mice were anesthetized with isoflurane supplemented with oxygen during the whole procedure. PET images were reconstructed as described for ^18^F-FDG-PET/MRI.

### Image Analysis

Positron emission tomography images were analyzed using PMOD v3.9 (PMOD Technologies, Switzerland) as previously described (16). In brief, different volumes of interest (VOI), including whole brain volume as well as the amygdala, brain stem, cerebellum, cortex, hippocampus, hypothalamus, midbrain, olfactory bulb, septum/basal forebrain, striatum, and thalamus were defined using an MRI-based mouse brain atlas template. VOI were between 10 mm^3^ (amygdala) and 0.3 cm^3^ (cortex) in size. VOI statistics (kBq/cc) were generated for all brain areas mentioned above and standardized uptake values (SUV) were calculated [SUV = tissue activity concentration average (kBq/cc) × body weight (g)/ injected dose (kBq)] for semiquantitative analysis. SUV of ^18^F-FDG-PET scans were corrected for measured blood glucose levels [Glc = SUV × blood glucose level (mg/dl)]. Blood glucose levels were between 116 and 209 mg/dl (mean 156 mg/dl) with no significant difference between the groups. ^18^F-Florbetaben—PET scans were normalized to the cerebellum, and the obtained ratios (SUVr) were used for further analysis.

### Behavior Experiments: Learning and Memory Performance

Animals (*n* = 10–12 per group) were kept on an inverted 12-h/12-h dark/light cycle and tested during the dark period.

#### Morris Water Maze

The Morris water maze (MWM) test was used to assess spatial reference memory as previously described (17). During the final “probe trial,” the platform was removed from the pool, and the mice were allowed to swim freely for 1 min while their swimming path and swimming speed were recorded using an automated video tracking system (ANY-Maze, Stoelting Co, USA).

#### Cross Maze

The cross maze was used to assess the working memory by analyzing the spontaneous alternation behavior of mice (18). The cross maze consists of four arms (30 cm length × 8 cm width × 15 cm height) arranged in 90° angles extending from a central region (8 cm length × 8 cm width × 15 cm height). During a single trial, mice were allowed to move freely through the maze for 10 min. ANY-Maze video tracking software (Stoelting Co, USA) was used to record the sequence of arm entries and the distance traveled. An alternation was defined as successive entries into the four arms in overlapping quadruple sets (e.g., 1, 3, 2, 4 or 2, 3, 4, 1 but not 1, 2, 3, 1) (15, 19). The alternation percentage was calculated as the percentage of actual alternations to the possible number of arm entries.

#### Novel Object Recognition

The novel object recognition task (NORT) is widely used to assess recognition memory and novelty preference in rodents. The test is based on the spontaneous tendency of rodents to explore a novel object more often than a familiar one (20). The NORT was performed in an open-field arena made of gray plastic (50 × 50 cm), and the animals were tracked using an automated video tracking system (ANY-maze, Stoelting Co, USA). A habituation phase 24 h before the actual test preceded the testing to avoid neophobic interference. During the habituation phase, each mouse was given 5 min to explore the testing arena and become habituated to the testing environment. Twenty-four hours after the habituation, animals were placed in the same box, containing two identical objects, and allowed to freely explore the box for 5 min. The following day, mice were placed back in the same arena, now with a familiar and novel object. The time spent with each object was recorded.

### Statistical Analysis

Statistical analysis was performed using GraphPad Prism version 8 for Mac (GraphPad Software, San Diego, CA, USA). Differences between groups were tested with a paired *t*-test, unpaired *t*-test, or one-way analysis of variance (ANOVA) followed by Bonferroni multiple comparisons as indicated. Data are given as mean +/– SD. Significance levels are given as follows: ^*^p < 0.05; ^**^p < 0.01; ^***^p < 0.0001.

## Results

### Female 5XFAD Mice Show Decreased Cerebral Glucose Metabolism Compared to WT Mice

**Figure 1:**
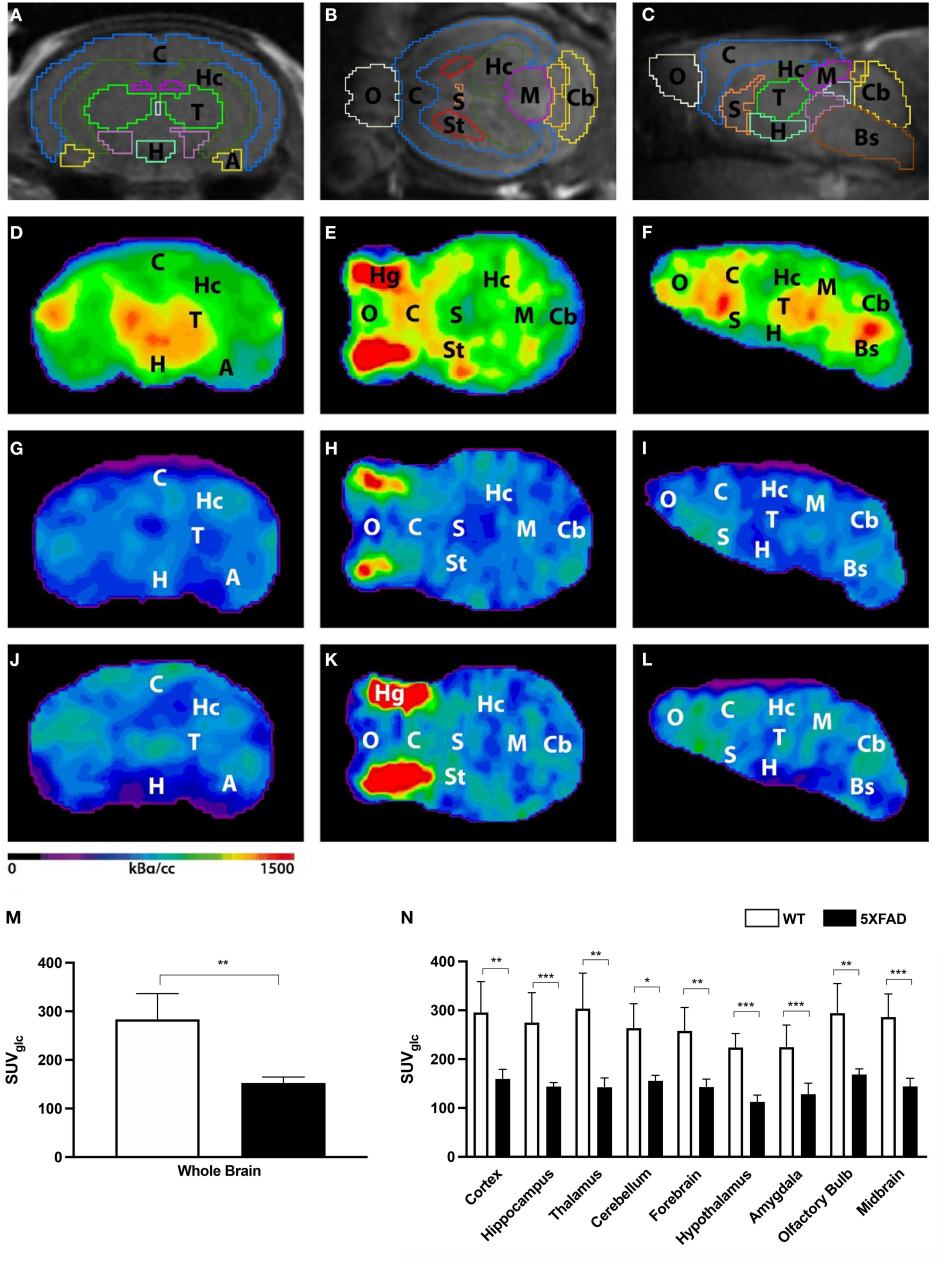
18F-FDG uptake in 7-month-old female 5XFAD mice and age- and sex-matched WT mice. **(A–C)** Mouse brain atlas with predefined brain regions projected on MRI images in coronal, transverse, and sagittal view. **(D–F)** Exemplary 18F-FDG-PET images of a 7-month-old female WT mouse. **(G–I)** 18F-FDG-PET images of a 7-month-old female 5XFAD mouse. **(J–L)** 18F-FDG-PET images of a 7-month-old male 5XFAD mouse. Seven-month-old female and male 5XFAD mice show significantly lower 18F-FDG uptake compared to WT mice. **(M)** 7-month-old female 5XFAD mice showed significantly lower SUVglc in the whole brain compared to 7-month-old female WT mice. **(N)** Differences could be detected in all brain regions. A, Amygdala; Bs, Brain Stem; C, Cortex; Cb, Cerebellum, H, Hypothalamus; Hc, Hippocampus; Hg, Harderian Glands; M, Midbrain; O, Olfactory Bulb; S, Septum/Basal Forebrain; St, Striatum; T, Thalamus. Unpaired *t*-test; *p < 0.05, **p < 0.01, ***p < 0.001; data presented as mean +/- SD.

Seven-month-old female 5XFAD and age- and sex-matched WT mice were scanned with ^18^F-FDG-PET to determine cerebral glucose metabolism *in vivo*. ^18^F-FDG uptake was measured in the whole brain VOI, and several brain region VOIs and glucose correction were performed (Figures 1A–L). ^18^F-FDG uptake was detected in the brain of all mice and also regular extracranial uptake within Harderian glands, myocardium, brown adipose tissue, intestines, kidneys, and the urinary bladder.

Seven-month-old female 5XFAD mice showed significantly lower ^18^F-FDG uptake in the whole brain compared with WT mice (Figure 1M, unpaired *t*-test: *p* = 0.0015). Decreased ^18^F-FDG uptake was detected within all brain regions (Figure 1N, unpaired *t*-test: cortex: *p* = 0.0035; hippocampus: *p* < 0.0001; thalamus: *p* = 0.0029; cerebellum: *p* = 0.0032; forebrain: *p* = 0.002; hypothalamus: *p* < 0.0001; amygdala: *p* < 0.0001; olfactory bulb: *p* = 0.004; midbrain: *p* < 0.0001).

### Female 5XFAD Mice Show Increased Amyloid Deposition Compared to WT Mice

**Figure 2:**
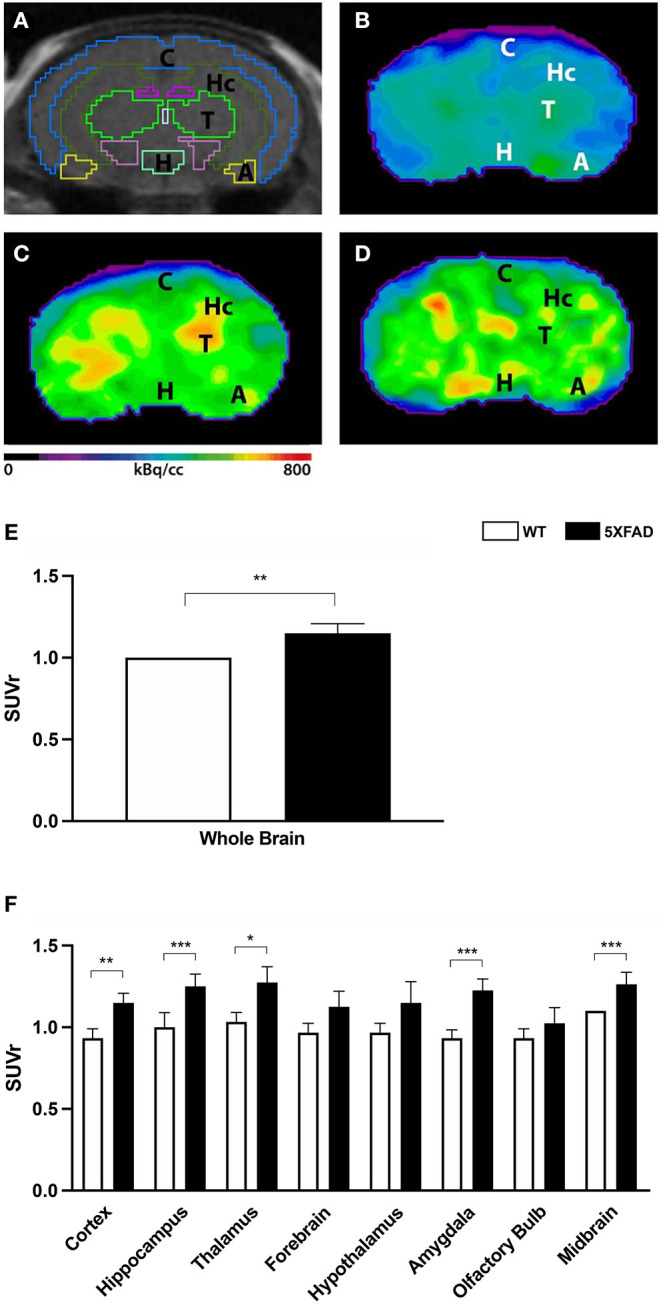
18F-Florbetaben uptake in 7-month-old female 5XFAD mice and age- and sex-matched WT mice. **(A)** Mouse brain atlas with predefined brain regions projected on MRI images in coronal view. **(B)** 18F-Florbetaben-PET images of a 7-month-old WT mouse. **(C)** 18F-Florbetaben–PET images of a 7-month-old female 5XFAD mouse. **(D)** 18F-Florbetaben-PET images of a 7-month-old male 5XFAD mouse. 18F-Florbetaben-PET detected increased amyloid deposition in 7-month-old female and male 5XFAD mice compared to WT. **(E)** 7-month-old female 5XFAD mice showed significantly higher SUVr in the whole brain compared with 7-month-old female WT mice. **(F)** Differences could be detected in the cortex, hippocampus, thalamus, amygdala, and midbrain. Unpaired *t*-test; *p < 0.05; **p < 0.01, ***p < 0.001; data presented as mean +/- SD.

To determine amyloid plaque deposition *in vivo*, 7-month-old 5XFAD mice and age- and sex-matched WT mice were examined with amyloid–PET using ^18^F-Florbetaben (Figures 2A–D). Tracer uptake was measured in the whole brain and eight different brain areas. ^18^F-Florbetaben uptake was normalized by cerebellar uptake as the reference region. ^18^F-Florbetaben was detected in the brain in all tested mice. Extracranial distribution was physiological, showing ^18^F-Florbetaben uptake within Harderian glands, intestines, and urinary bladder.

Female 5XFAD mice showed significantly increased ^18^F-Florbetaben uptake compared with same-aged WT animals in the whole brain (Figure 2E, unpaired *t*-test: *p* = 0.0071) as well as the cortex (Figure 2F, unpaired *t*-test: *p* = 0.0044), hippocampus (*p* < 0.0001), thalamus (*p* = 0.0123), amygdala (*p* < 0.0001), and midbrain (*p* = 0.0002).

### Female 5XFAD Mice Show Lower Cerebral Glucose Metabolism Compared to Male 5XFAD Mice

**Figure 3:**
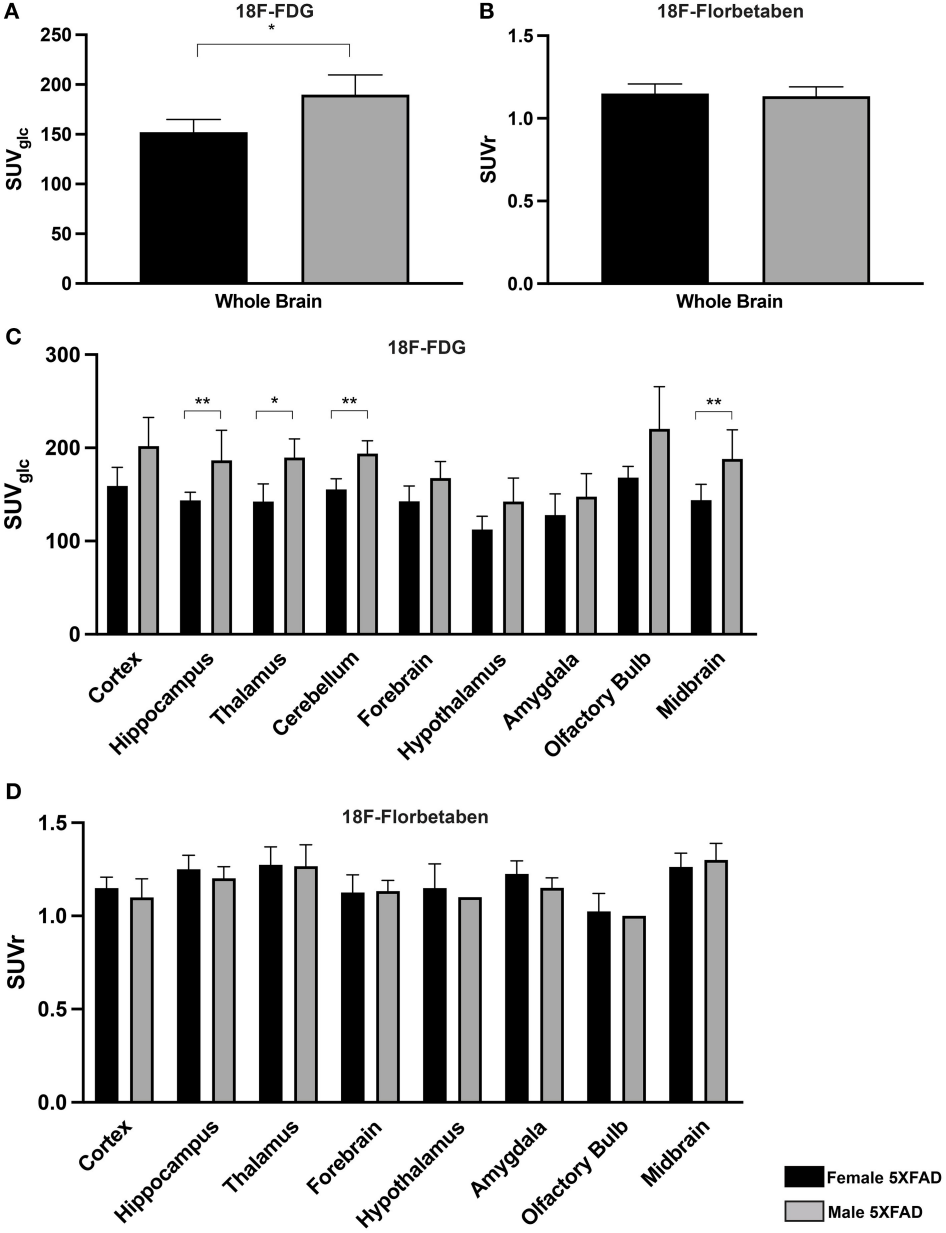
Sex differences in 7-month-old 5XFAD mice. **(A,C)** 18F-FDG uptake in 7-month-old female 5XFAD mice showed significantly lower SUVglc in the whole brain compared to 7-month-old male 5XFAD mice. Differences could be detected in the hippocampus, thalamus, cerebellum, and midbrain. **(B)** 7-month-old female 5XFAD mice did not show significant differences in 18F-Florbetaben uptake compared to 7-month-old male 5XFAD mice in the whole brain or in **(D)** any of the analyzed brain regions. Unpaired *t*-test; *p < 0.05; **p < 0.01; data presented as mean +/- SD.

To determine sex differences in cerebral glucose metabolism of 5XFAD mice, ^18^F-FDG-PET results of 7-month-old female 5XFAD and age-matched male 5XFAD mice were compared. Whole brain ^18^F-FDG uptake was significantly lower in female 5XFAD mice (Figure 3A, unpaired *t*-test: *p* = 0.0286). Differences were detected in the hippocampus (Figure 3C, *t*-test: *p* = 0.0035), thalamus (*p* = 0.0245), cerebellum (*p* = 0.0097), and midbrain (*p* = 0.005).

### Amyloid Deposition in ^18^F-Florbetaben-PET Did Not Differ Between Female and Male 5XFAD Mice

Seven-month-old female 5XFAD and age-matched male 5XFAD mice did not show significant differences in ^18^F-Florbetaben uptake in the whole brain and all tested brain areas (Figures 3B,D, unpaired *t*-test: whole brain: *p* = 0.721; cortex: *p* = 0.4366; hippocampus: *p* = 0.2149; thalamus: *p* = 0.9206; forebrain: *p* = 0.9001; hypothalamus: *p* = 0.5416; amygdala: *p* = 0.0525; olfactory bulb: *p* = 0.6774; midbrain: *p* = 0.4082).

### Memory Deficits in Female 7-Month-Old 5XFAD Mice

**Figure 4:**
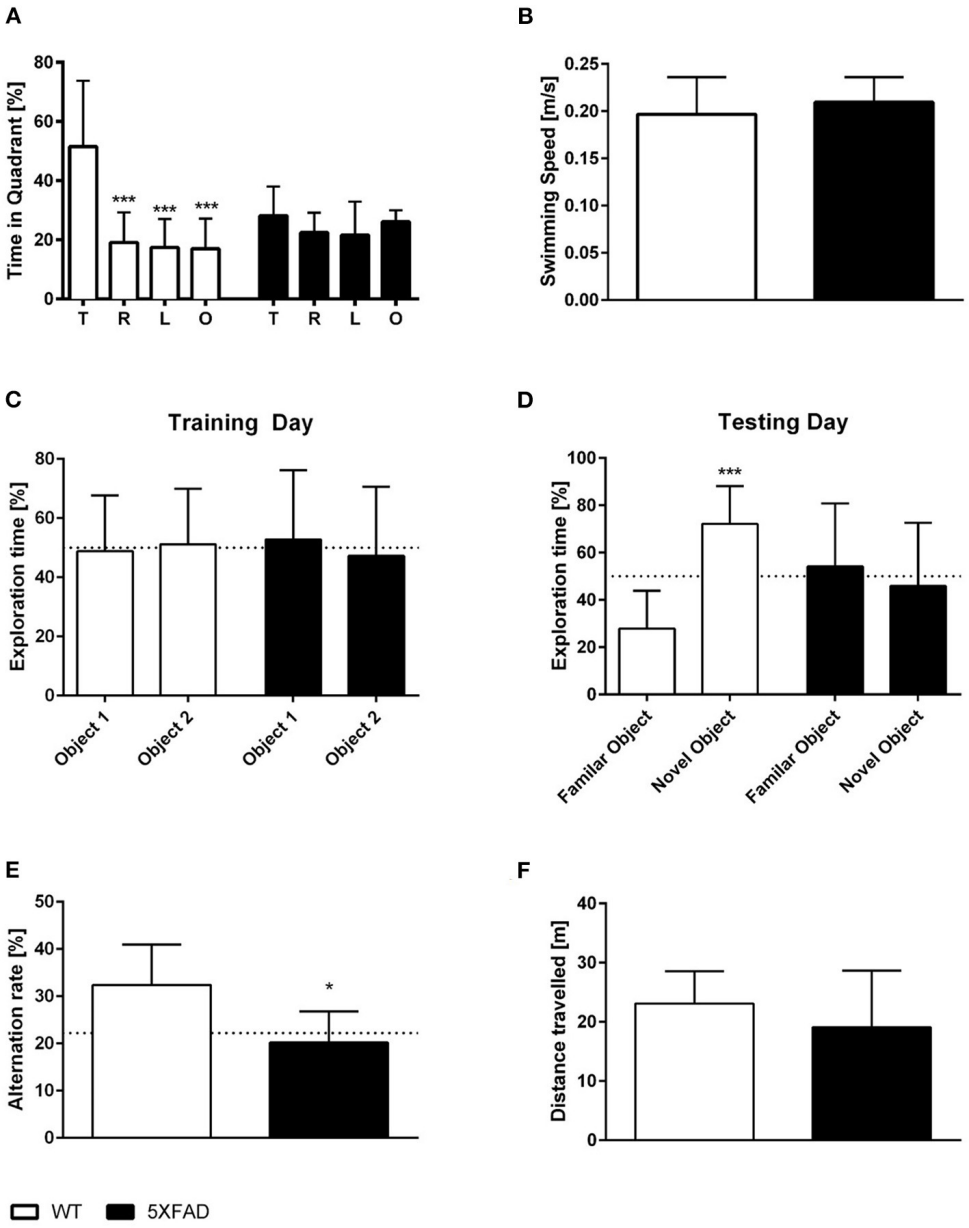
Memory deficits in female 7-month-old 5XFAD mice. **(A)** 5XFAD mice displayed an impaired spatial reference memory in the probe trial of the Morris water maze as they displayed no preference for the target quadrant. In contrast, WT animals showed a clear preference for the target quadrant. **(B)** Swimming speed did not differ between WT and 5XFAD mice. **(C,D)** In addition, 5XFAD mice displayed an impaired objection memory. **(C)** During the exploration phase, both genotypes spent ~ 50% of their time exploring each of the two similar objects. **(D)** However, only WT mice showed a preference for the novel object on the testing day. **(E)** Impaired working memory in 5XFAD mice demonstrated in the cross maze (dotted line indicates chance level). **(F)** There was no difference in the total distance traveled between 5XFAD and WT mice in the cross maze. **(A)** One-way ANOVA followed by Bonferroni multiple comparisons **(B,E,F)** unpaired *t*-test, **(C,D)** paired *t*-test. *p < 0.05; ***p < 0.001; Data presented as mean ± SD.

Spatial reference memory was assessed in female 5XFAD and WT mice in the probe trial of the Morris water maze. WT mice displayed a significant preference for the target quadrant as indicated by the percentage time spent in the different quadrants of the pool (Figure 4A, One-way ANOVA, WT: *p* < 0.001 targets vs. all other quadrants). In contrast, 5XFAD mice showed no preference for any of the quadrants (Figure 4A). The absence of a preference for the target quadrant compared to the remaining quadrants demonstrates that 5XFAD mice display a deficit in spatial reference memory. Swimming speed was not altered in 5XFAD mice (Figure 4B, unpaired *t*-test: *p* = 0.3651).

Recognition memory was tested using the NORT. Female 7-month-old 5XFAD mice showed an impaired recognition memory as they displayed no preference for the novel object (Figure 4D, paired *t*-test, 5XFAD: *p* = 0.6999). In contrast, WT animals showed a clear preference for the novel object and intact recognition memory (Figure 4D, paired *t*-test, WT: *p* < 0.0001). During the training phase, WT and 5XFAD spend ~50% of the time with both identical objects (Figure 4C, paired *t*-test, WT: *p* = 0.8355; 5XFAD: *p* = 0.7825).

In addition, female 5XFAD mice showed an impaired spatial working memory in the cross maze. The alternation rate was used as an indicator of spatial working memory impairment in this task, and 5XFAD mice displayed a significantly reduced alternation rate compared with the same-aged wild type animals (Figure 4E, unpaired *t*-test, *p* = 0.0112). The reduced alternation rate was not due to a decrease in overall exploration behavior as mice covered the same distance (Figure 4F, unpaired *t*-test, *p* = 0.3207).

## Discussion

Alzheimer's disease is one of the most challenging diseases of the century. To investigate new therapies, longitudinal evaluation of their effects *in vivo* is essential and relies heavily on disease-specific biomarkers. Biomarkers, including molecular imaging with ^18^F-FDG- and amyloid-PET, have shown their value in the clinical diagnosis of AD (21). However, only a limited number of preclinical studies with PET are available, even though modern small animal PET scanners are able to detect the same molecular pathologies that are seen in humans. Both, ^18^F-FDG– and amyloid–PET can detect early changes of the disease, making them valuable targets for *in vivo* imaging of disease progression and efficacy of new therapeutic strategies. Advances in small animal PET have enabled imaging in AD mouse models, allowing longitudinal follow-up studies (22, 23) and a better translation of findings from bench to bedside.

Neuronal dysfunctions can be detected using ^18^F-FDG–PET as ^18^F-FDG uptake in neurons is mainly determined by synaptic activity (24). AD patients typically show a pattern of decreased ^18^F-FDG uptake in the posterior cingulate cortex proceeding to posterior temporal and parietal cortex areas and eventually to the frontal lobe (25). However, the use of ^18^F-FDG as a preclinical biomarker to study AD processes in animal models has been arguable, as inconsistent results with hyper-, hypo-, and normal metabolism have been described in transgenic AD mouse models (12, 26–28).

The 5XFAD mouse model of AD is a widely used model of amyloidosis with five AD-linked mutations. 5XFAD mice show a massive plaque pathology, intraneuronal Abeta, robust microgliosis, and inflammatory processes as well as synaptic and neuronal loss and memory deficits (9, 10, 15, 29, 30). Unbiased stereology revealed significant neuron loss in the subiculum by 9 months and neuron layer 5 of the cortex by 12 months in female 5XFAD mice (15, 29). The robust appearance of histological, molecular, and behavioral AD hallmarks makes the 5XFAD model popular in biomedical research, and the “Alzheimer's Disease Preclinical Efficacy Database” (https://alzped.nia.nih.gov/) shows that ~10% of all AD studies that work with animal models use this strain. Therefore, it is essential that reliable biomarkers are available to monitor the success of a possible therapy in 5XFAD mice. As 5XFAD mice recapitulate major features of AD, ^18^F-FDG– and amyloid-PET should theoretically be useful *in vivo* biomarkers for 5XFAD animals. However, only a few PET studies with inconsistent findings, which largely focus on male mice, have been performed in 5XFAD mice (11, 13, 31, 32). The current study, which is the first preclinical study using PET/MRI with different tracers in female 5XFAD mice, showed distinct reduced ^18^F-FDG uptake in the whole brain as well as in all the nine analyzed brain regions including the hippocampus, cortex, thalamus, and hypothalamus. To our knowledge, only one other study analyzed cerebral glucose metabolism in female 5XFAD mice (33). Son et al. focused on the hippocampus and frontal lobe and described a regional hypometabolism in these two brain regions in 9.5-month-old female 5XFAD mice. In line with these findings, Xiao et al. (34) demonstrated decreased ^18^F-FDG uptake in the hippocampus, cerebral cortex, and olfactory bulb of 6-month-old male 5XFAD mice. Similarly, DeBay et al. (32) confirmed a hypometabolism in the cerebral cortex, hippocampus, and basal ganglia in 5-month-old male 5XFAD mice. In addition, Macdonald et al. (13) detected lower ^18^F-FDG uptake in the whole brain of 13-month-old male 5XFAD mice. In contrast, a study by Rojas et al. (31) showed contradictory findings with increased ^18^F-FDG uptake in the whole brain of 11-month-old 5XFAD mice, without stating the sex, using the cerebellum as the reference region. As discussed before (11), this normalization technique seems to heavily influence study results. In contrast to human glucose metabolism in the cerebellum (which is relatively preserved in disease progression), metabolism in the cerebellum of transgenic mouse models can also be influenced, possibly resulting in higher uptake values (27, 35–37). Studies in other AD mouse models using normalized cerebral FDG uptake to the cerebellum also showed higher ^18^F-FDG uptake in the brain (27, 36, 37). This factor could explain the results by Rojas et al. (31) in 5XFAD mice.

To our knowledge, no studies on sex differences in ^18^F-FDG-PET in 5XFAD mice or other transgenic mouse models of AD are available so far. We could previously demonstrate a significant reduction in glucose metabolism in 7- and 12-month-old male 5XFAD mice in several cortical areas even before the onset of memory defecits (11). In this study, we show for the first time that the hypometabolism in 5XFAD mice is gender-dependent and more pronounced in female mice. Our results demonstrated a lower glucose metabolism in the whole brain as well as in the hippocampus, thalamus, cerebellum, and midbrain of 7-month-old female 5XFAD mice compared with age-matched male 5XFAD mice.

Although the reason is still unknown, AD has a gender-specific epidemiological profile, disproportionately affecting women both in prevalence and in severity (38, 39). A number of female transgenic AD mouse models also display an earlier onset of AD pathologies as well as a more severe pathology than their male counterparts (40–42). Moreover, behavior deficits are often more severe in female mice (43, 44). Sex-based differences in 5XFAD mice have also been demonstrated in several studies ranging from differences in odor detection and motor impairment to working memory deficits (45–47). A gene ontology analysis of the hippocampi of 4-month-old 5XFAD mice detected sex-specific patterns with female 5XFAD mice, which showed more differentially expressed genes compared with males including higher levels of human APP and PS1 mRNA expression. Interestingly, the majority of molecular changes that were more severely affected in female 5XFAD mice were associated with the immune system (48).

Another pathological hallmark of AD that can be detected by PET is cerebral amyloid depositions. Several PET-tracers that are able to visualize amyloid burden have been developed and are used in clinical routine including ^11^C-labeled Pittsburgh Compound-B (^11^C-PIB), ^18^F-Flutemetamol, ^18^F-Florbetapir, and ^18^F-Florbetaben (21, 49–52). Amyloid–PET has also been demonstrated as a valuable tool in preclinical studies and can be used to measure therapeutic outcomes *via* amyloid load *in vivo* (53–56). The presence of a distinctive plaque pathology in 5XFAD mice has been well-described *in vitro* with extracellular amyloid plaques beginning at 2 months in the fifth layer of the cortex and progressively increasing with age and spreading to the cortex, hippocampus, and subiculum (9, 15, 57). However, only a few studies on the use of amyloid–PET in 5XFAD mice are available so far, lacking data on female 5XFAD mice. Available studies show higher cerebral uptake of several different amyloid tracers (^11^C-PIB, ^18^F-Florbetapir,^18^F-FC119S, and ^18^F-Florbetaben) compared with WT mice. Rojas and colleagues reported a higher uptake of the amyloid radiotracers ^11^C-PIB and ^18^F-Florbetapir in 11-month-old 5XFAD mice, whereas Frost et al. showed elevated uptake of ^18^F-Florbetapir in 14-month-old mice, both without stating the sex (31, 58). In addition, Oh et al. demonstrated elevated levels of ^18^F-FC119S in the cortex, hippocampus, and thalamus of 5.5-month-old male 5XFAD mice (14). We previously showed an increased ^18^F-Florbetaben tracer uptake in male 5XFAD mice (11). Here we could confirm that ^18^F-Florbetaben is also a valuable tracer for female 5XFAD as our findings demonstrate increased cerebral uptake of ^18^F-Florbetaben in 7-month-old female 5XFAD mice.

In contrast to ^18^F-FDG uptake, ^18^F-Florbetaben–PET did not show significant sex-differences between male and female 5XFAD mice. This is in line with the original manuscript characterizing the 5XFAD model, as Oakley et al. only noted a trend toward greater plaque deposition in young female AD animals relative to males of the same age, but this trend declined with age (9). In contrast, several studies demonstrated higher levels of APP and Aβ42 as well as an increased plaque pathology in female 5XFAD mice (48, 59, 60). In line with our findings, Biechele and colleagues also detected no sex differences in ^18^F-Florbetaben uptake in App^NL−G−F^ mice (61).

Limitations of our study include possible partial volume effect due to analysis of relatively small VOIs. However, this effect might have been avoided as the analyzed volumes were above the suggested threshold in small animal scanners of 9 mm^3^ [the smallest VOI (amygdala) was 10 mm^3^] (62–64). Furthermore, there is no standard for small animal brain imaging which can result in high inter-study variations, especially for FDG studies. Therefore, to avoid inconsistencies between different labs, standardization of imaging protocols including fasting protocols, equipment, and animal handling would be beneficial (12, 65). In addition, it might be interesting to study additional age groups for female 5XFAD mice in the future. However, we decided to focus on 7-month-old mice as female 5XFAD mice show a robust Abeta pathology as well as severe memory deficits at this age. Furthermore, this age is often used as an endpoint in treatment studies in 5XFAD mice (66–71). Thus, our present findings underline that PET imaging with ^18^F-FDG- and ^18^F-Florbetaben could provide a valuable and robust preclinical evaluation tool of therapeutic strategies modulating the AD pathology in 5XFAD mice.

Our results support the feasibility of small animal PET imaging with ^18^F-FDG- and ^18^F-Florbetaben in the 5XFAD mouse model of AD in both male and female animals. Moreover, our findings highlight the need to account for sex differences in studies working with 5XFAD mice.

## Data Availability Statement

The raw data supporting the conclusions of this article will be made available by the authors, without undue reservation.

## Ethics Statement

The animal study was reviewed and approved by Niedersächsisches Landesamt für Verbraucherschutz und Lebensmittelsicherheit, Röverskamp 5, 26203 Oldenburg, Germany and Landesamt für Gesundheit und Soziales LAGeSo Darwinstr. 15, 10589 Berlin, Germany.

## Author Contributions

TF, CI, and NB performed experiments. CB and YB designed the project, performed experiments, analyzed data, and wrote the manuscript. All authors contributed to revising the manuscript and approved the final version.

## Funding

This work was supported by the Alzheimer Stiftung Göttingen to CB and YB, and in part by the Deutsche Forschungsgemeinschaft (DFG) for PET/MRI use (INST 335/454-1FUGG). We acknowledge support by the Open Access Publication Funds of the Göttingen University.

## Conflict of Interest

The authors declare that the research was conducted in the absence of any commercial or financial relationships that could be construed as a potential conflict of interest.

## Publisher's Note

All claims expressed in this article are solely those of the authors and do not necessarily represent those of their affiliated organizations, or those of the publisher, the editors and the reviewers. Any product that may be evaluated in this article, or claim that may be made by its manufacturer, is not guaranteed or endorsed by the publisher.

## References

[1] Alzheimer's Disease International. World Alzheimer Report 2018 - The State of the Art Dementia Research: New Frontiers London: Alzheimer's Disease International. (2018). Available online at: https://www.alz.co.uk/news/world-alzheimer-report-2018-state-of-art-of-dementia-research-new-frontiers (accessed May 01, 2021).

[2] Anand A Patience AA Sharma N Khurana N. The present and future of pharmacotherapy of Alzheimer's disease: a comprehensive review. Eur J Pharmacol. (2017) 815:364–75. 10.1016/j.ejphar.2017.09.043 28978455

[3] Kishi T Sakuma K Iwata N. Efficacy and safety of psychostimulants for Alzheimer's disease: a systematic review and meta-analysis. Pharmacopsychiatry. (2020) 53:109–14. 10.1055/a-1076-8228 32000270

[4] Del Sole A Malaspina S Magenta Biasina A. Magnetic resonance imaging and positron emission tomography in the diagnosis of neurodegenerative dementias. Funct Neurol. (2016) 31:205–15. 10.11138/FNeur/2016.31.4.205 28072381 PMC5231883

[5] Librizzi D Cabanel N Zavorotnyy M Riehl E Kircher T Luster M. Clinical Relevance of [(18)F]Florbetaben and [(18)F]FDG PET/CT imaging on the management of patients with dementia. Molecules. (2021) 26:1282. 10.3390/molecules26051282 33652938 PMC7956266

[6] Ferrari C Caputo P Pisani AR Nappi AG Branca A Lavelli V. Use of amyloid PET/CT with (18)F-Florbetaben in the management of patients with Alzheimer's disease. Hell J Nucl Med. (2019) 22 (Suppl 2):142–52. 31802055

[7] Ou YN Xu W Li JQ Guo Y Cui M Chen KL. FDG-PET as an independent biomarker for Alzheimer's biological diagnosis: a longitudinal study. Alzheimers Res Ther. (2019) 11:57. 10.1186/s13195-019-0512-1 31253185 PMC6599313

[8] Villemagne VL Ong K Mulligan RS Holl G Pejoska S Jones G. Amyloid imaging with (18)F-florbetaben in Alzheimer disease and other dementias. J Nucl Med. (2011) 52:1210–7. 10.2967/jnumed.111.089730 21764791

[9] Oakley H Cole SL Logan S Maus E Shao P Craft J. Intraneuronal beta-amyloid aggregates, neurodegeneration, and neuron loss in transgenic mice with five familial Alzheimer's disease mutations: potential factors in amyloid plaque formation. J Neurosci. (2006) 26:10129–40. 10.1523/JNEUROSCI.1202-06.2006 17021169 PMC6674618

[10] Bouter Y Kacprowski T Weissmann R Dietrich K Borgers H Brauss A. Deciphering the molecular profile of plaques, memory decline and neuron loss in two mouse models for Alzheimer's disease by deep sequencing. Front Aging Neurosci. (2014) 6:75. 10.3389/fnagi.2014.00075 24795628 PMC3997018

[11] Franke TN Irwin C Bayer TA Brenner W Beindorff N Bouter C. *In vivo* imaging with (18)F-FDG- and (18)F-Florbetaben-PET/MRI detects pathological changes in the brain of the commonly used 5XFAD mouse model of Alzheimer's disease. Front Med. (2020) 7:529. 10.3389/fmed.2020.00529 33043029 PMC7522218

[12] Bouter C Bouter Y. (18)F-FDG-PET in mouse models of Alzheimer's disease. Front Med. (2019) 6:71. 10.3389/fmed.2019.00071 31058151 PMC6482246

[13] Macdonald IR DeBay DR Reid GA O'Leary TP Jollymore CT Mawko G. Early detection of cerebral glucose uptake changes in the 5XFAD mouse. Curr Alzheimer Res. (2014) 11:450–60. 10.2174/1567205011666140505111354 24801216 PMC4082185

[14] Oh SJ Lee HJ Kang KJ Han SJ Lee YJ Lee KC. Early detection of abeta deposition in the 5xFAD mouse by Amyloid PET. Contrast Media Mol Imaging. (2018) 2018:5272014. 10.1155/2018/5272014 29681782 PMC5851318

[15] Jawhar S Trawicka A Jenneckens C Bayer TA Wirths O. Motor deficits, neuron loss, and reduced anxiety coinciding with axonal degeneration and intraneuronal Abeta aggregation in the 5XFAD mouse model of Alzheimer's disease. Neurobiol Aging. (2012) 33:196 e29–40. 10.1016/j.neurobiolaging.2010.05.027 20619937

[16] Bouter C Henniges P Franke TN Irwin C Sahlmann CO Sichler ME. (18)F-FDG-PET detects drastic changes in brain metabolism in the Tg4-42 model of Alzheimer's disease. Front Aging Neurosci. (2018) 10:425. 10.3389/fnagi.2018.00425 30670962 PMC6333025

[17] Bouter Y Dietrich K Wittnam JL Rezaei-Ghaleh N Pillot T Papot-Couturier S. N-truncated amyloid beta (Abeta) 4-42 forms stable aggregates and induces acute and long-lasting behavioral deficits. Acta Neuropathol. (2013) 126:189–205. 10.1007/s00401-013-1129-2 23685882 PMC3722453

[18] Cleal M Fontana BD Ranson DC McBride SD Swinny JD Redhead ES. The Free-movement pattern Y-maze: a cross-species measure of working memory and executive function. Behav Res Methods. (2021) 53:536–57. 10.3758/s13428-020-01452-x 32748238 PMC8062322

[19] Arendash GW King DL Gordon MN Morgan D Hatcher JM Hope CE. Progressive, age-related behavioral impairments in transgenic mice carrying both mutant amyloid precursor protein and presenilin-1 transgenes. Brain Res. (2001) 891:42–53. 10.1016/S0006-8993(00)03186-3 11164808

[20] Grayson B Leger M Piercy C Adamson L Harte M Neill JC. Assessment of disease-related cognitive impairments using the novel object recognition (NOR) task in rodents. Behav Brain Res. (2015) 285:176–93. 10.1016/j.bbr.2014.10.025 25447293

[21] Chetelat G Arbizu J Barthel H Garibotto V Law I Morbelli S. Amyloid-PET and (18)F-FDG-PET in the diagnostic investigation of Alzheimer's disease and other dementias. Lancet Neurol. (2020) 19:951–62. 10.1016/S1474-4422(20)30314-8 33098804

[22] Maeda J Ji B Irie T Tomiyama T Maruyama M Okauchi T. Longitudinal, quantitative assessment of amyloid, neuroinflammation, and anti-amyloid treatment in a living mouse model of Alzheimer's disease enabled by positron emission tomography. J Neurosci. (2007) 27:10957–68. 10.1523/JNEUROSCI.0673-07.2007 17928437 PMC6672864

[23] Takkinen JS Lopez-Picon FR Al Majidi R Eskola O Krzyczmonik A Keller T. Brain energy metabolism and neuroinflammation in ageing APP/PS1-21 mice using longitudinal (18)F-FDG and (18)F-DPA-714 PET imaging. J Cereb Blood Flow Metab. (2017) 37:2870–82. 10.1177/0271678X16677990 27834284 PMC5536795

[24] Attwell D Laughlin SB. An energy budget for signaling in the grey matter of the brain. J Cereb Blood Flow Metab. (2001) 21:1133–45. 10.1097/00004647-200110000-00001 11598490

[25] Marcus C Mena E Subramaniam RM. Brain PET in the diagnosis of Alzheimer's disease. Clin Nucl Med. (2014) 39:e413–22; quiz e23–6. 10.1097/RLU.0000000000000547 25199063 PMC4332800

[26] Kuntner C Kesner AL Bauer M Kremslehner R Wanek T Mandler M. Limitations of small animal PET imaging with [18F]FDDNP and FDG for quantitative studies in a transgenic mouse model of Alzheimer's disease. Mol Imaging Biol. (2009) 11:236–40. 10.1007/s11307-009-0198-z 19214638

[27] Brendel M Probst F Jaworska A Overhoff F Korzhova V Albert NL. Glial activation and glucose metabolism in a transgenic amyloid mouse model: a triple-tracer PET study. J Nucl Med. (2016) 57:954–60. 10.2967/jnumed.115.167858 26912428

[28] Coleman RA Liang C Patel R Ali S Mukherjee J. Brain and brown adipose tissue metabolism in transgenic Tg2576 mice models of Alzheimer disease assessed using (18)F-FDG PET imaging. Mol Imaging. (2017) 16:1536012117704557. 10.1177/1536012117704557 28654383 PMC5470140

[29] Eimer WA Vassar R. Neuron loss in the 5XFAD mouse model of Alzheimer's disease correlates with intraneuronal Abeta42 accumulation and Caspase-3 activation. Mol Neurodegener. (2013) 8:2. 10.1186/1750-1326-8-2 23316765 PMC3552866

[30] Buskila Y Crowe SE Ellis-Davies GC. Synaptic deficits in layer 5 neurons precede overt structural decay in 5xFAD mice. Neuroscience. (2013) 254:152–9. 10.1016/j.neuroscience.2013.09.016 24055684 PMC4078998

[31] Rojas S Herance JR Gispert JD Abad S Torrent E Jimenez X. *In vivo* evaluation of amyloid deposition and brain glucose metabolism of 5XFAD mice using positron emission tomography. Neurobiol Aging. (2013) 34:1790–8. 10.1016/j.neurobiolaging.2012.12.027 23402900

[32] DeBay DR Reid GA Macdonald IR Mawko G Burrell S Martin E. Butyrylcholinesterase-knockout reduces fibrillar beta-amyloid and conserves (18)FDG retention in 5XFAD mouse model of Alzheimer's disease. Brain Res. (2017) 1671:102–10. 10.1016/j.brainres.2017.07.009 28729192

[33] Son Y Kim JS Jeong YJ Jeong YK Kwon JH Choi HD. Long-term RF exposure on behavior and cerebral glucose metabolism in 5xFAD mice. Neurosci Lett. (2018) 666:64–9. 10.1016/j.neulet.2017.12.042 29273398

[34] Xiao NA Zhang J Zhou M Wei Z Wu XL Dai XM. Reduction of glucose metabolism in olfactory bulb is an earlier Alzheimer's disease-related biomarker in 5XFAD Mice. Chin Med J. (2015) 128:2220–7. 10.4103/0366-6999.162507 26265617 PMC4717990

[35] Deleye S Waldron AM Richardson JC Schmidt M Langlois X Stroobants S. The effects of physiological and methodological determinants on 18F-FDG mouse brain imaging exemplified in a double transgenic Alzheimer model. Mol Imaging. (2016) 15:1536012115624919. 10.1177/1536012115624919 27030402 PMC5470082

[36] Poisnel G Herard AS El Tannir El Tayara N Bourrin E Volk A Kober F. Increased regional cerebral glucose uptake in an APP/PS1 model of Alzheimer's disease. Neurobiol Aging. (2012) 33:1995–2005. 10.1016/j.neurobiolaging.2011.09.026 22079157 PMC3666917

[37] Li XY Men WW Zhu H Lei JF Zuo FX Wang ZJ. Age- and brain region-specific changes of glucose metabolic disorder, learning, and memory dysfunction in early Alzheimer's disease assessed in APP/PS1 transgenic mice using (18)F-FDG-PET. Int J Mol Sci. (2016) 17:1707. 10.3390/ijms17101707 27763550 PMC5085739

[38] Carter CL Resnick EM Mallampalli M Kalbarczyk A. Sex and gender differences in Alzheimer's disease: recommendations for future research. J Womens Health. (2012) 21:1018–23. 10.1089/jwh.2012.3789 22917473

[39] Laws KR Irvine K Gale TM. Sex differences in cognitive impairment in Alzheimer's disease. World J Psychiatry. (2016) 6:54–65. 10.5498/wjp.v6.i1.54 27014598 PMC4804268

[40] Callahan MJ Lipinski WJ Bian F Durham RA Pack A Walker LC. Augmented senile plaque load in aged female beta-amyloid precursor protein-transgenic mice. Am J Pathol. (2001) 158:1173–7. 10.1016/S0002-9440(10)64064-3 11238065 PMC1850367

[41] Gallagher JJ Minogue AM Lynch MA. Impaired performance of female APP/PS1 mice in the Morris water maze is coupled with increased Abeta accumulation and microglial activation. Neurodegener Dis. (2013) 11:33–41. 10.1159/000337458 22627185

[42] Hirata-Fukae C Li HF Hoe HS Gray AJ Minami SS Hamada K. Females exhibit more extensive amyloid, but not tau, pathology in an Alzheimer transgenic model. Brain Res. (2008) 1216:92–103. 10.1016/j.brainres.2008.03.079 18486110

[43] King DL Arendash GW Crawford F Sterk T Menendez J Mullan MJ. Progressive and gender-dependent cognitive impairment in the APP(SW) transgenic mouse model for Alzheimer's disease. Behav Brain Res. (1999) 103:145–62. 10.1016/S0166-4328(99)00037-6 10513583

[44] Granger MW Franko B Taylor MW Messier C George-Hyslop PS Bennett SA. A TgCRND8 mouse model of Alzheimer's disease exhibits sexual dimorphisms in behavioral indices of cognitive reserve. J Alzheimers Dis. (2016) 51:757–73. 10.3233/JAD-150587 26890738

[45] Roddick KM Roberts AD Schellinck HM Brown RE. Sex and genotype differences in odor detection in the 3xTg-AD and 5XFAD mouse models of Alzheimer's disease at 6 months of age. Chem Senses. (2016) 41:433–40. 10.1093/chemse/bjw018 26969629

[46] O'Leary TP Mantolino HM Stover KR Brown RE. Age-related deterioration of motor function in male and female 5xFAD mice from 3 to 16 months of age. Genes Brain Behav. (2020) 19:e12538. 10.1111/gbb.12538 30426678

[47] Roddick KM Schellinck HM Brown RE. Olfactory delayed matching to sample performance in mice: sex differences in the 5XFAD mouse model of Alzheimer's disease. Behav Brain Res. (2014) 270:165–70. 10.1016/j.bbr.2014.04.038 24786328

[48] Bundy JL Vied C Badger C Nowakowski RS. Sex-biased hippocampal pathology in the 5XFAD mouse model of Alzheimer's disease: a multi-omic analysis. J Comp Neurol. (2019) 527:462–75. 10.1002/cne.24551 30291623

[49] Meyer PT Hellwig S Amtage F Rottenburger C Sahm U Reuland P. Dual-biomarker imaging of regional cerebral amyloid load and neuronal activity in dementia with PET and 11C-labeled Pittsburgh compound B. J Nucl Med. (2011) 52:393–400. 10.2967/jnumed.110.083683 21321269

[50] Barthel H Sabri O. Florbetaben to trace amyloid-beta in the Alzheimer brain by means of PET. J Alzheimers Dis. (2011) 26 (Suppl.3):117–21. 10.3233/JAD-2011-0068 21971456

[51] Tateno A Sakayori T Kawashima Y Higuchi M Suhara T Mizumura S. Comparison of imaging biomarkers for Alzheimer's disease: amyloid imaging with [18F]florbetapir positron emission tomography and magnetic resonance imaging voxel-based analysis for entorhinal cortex atrophy. Int J Geriatr Psychiatry. (2015) 30:505–13. 10.1002/gps.4173 25043833

[52] Zwan MD Bouwman FH Konijnenberg E van der Flier WM Lammertsma AA Verhey FR. Diagnostic impact of [(18)F]flutemetamol PET in early-onset dementia. Alzheimers Res Ther. (2017) 9:2. 10.1186/s13195-016-0228-4 28093088 PMC5240413

[53] Brendel M Jaworska A Herms J Trambauer J Rotzer C Gildehaus FJ. Amyloid-PET predicts inhibition of *de novo* plaque formation upon chronic gamma-secretase modulator treatment. Mol Psychiatry. (2015) 20:1179–87. 10.1038/mp.2015.74 26055427 PMC4759098

[54] Brendel M Jaworska A Overhoff F Blume T Probst F Gildehaus FJ. Efficacy of chronic BACE1 inhibition in PS2APP mice depends on the regional Abeta deposition rate and plaque burden at treatment initiation. Theranostics. (2018) 8:4957–68. 10.7150/thno.27868 30429879 PMC6217065

[55] Snellman A Rokka J Lopez-Picon FR Helin S Re F Loyttyniemi E. Applicability of [(11)C]PIB micro-PET imaging for *in vivo* follow-up of anti-amyloid treatment effects in APP23 mouse model. Neurobiol Aging. (2017) 57:84–94. 10.1016/j.neurobiolaging.2017.05.008 28605642

[56] Cheng YS Chen ZT Liao TY Lin C Shen HC Wang YH. An intranasally delivered peptide drug ameliorates cognitive decline in Alzheimer transgenic mice. EMBO Mol Med. (2017) 9:703–15. 10.15252/emmm.201606666 28356312 PMC5412883

[57] Kim DK Han D Park J Choi H Park JC Cha MY. Deep proteome profiling of the hippocampus in the 5XFAD mouse model reveals biological process alterations and a novel biomarker of Alzheimer's disease. Exp Mol Med. (2019) 51:1–17. 10.1038/s12276-019-0326-z 31727875 PMC6856180

[58] Frost GR Longo V Li T Jonas LA Judenhofer M Cherry S. Author correction: hybrid PET/MRI enables high-spatial resolution, quantitative imaging of amyloid plaques in an Alzheimer's disease mouse model. Sci Rep. (2020) 10:13826. 10.1038/s41598-020-70134-7 32778663 PMC7417542

[59] Sadleir KR Eimer WA Cole SL Vassar R. Abeta reduction in BACE1 heterozygous null 5XFAD mice is associated with transgenic APP level. Mol Neurodegener. (2015) 10:1. 10.1186/1750-1326-10-1 25567526 PMC4297413

[60] Bhattacharya S Haertel C Maelicke A Montag D. Galantamine slows down plaque formation and behavioral decline in the 5XFAD mouse model of Alzheimer's disease. PLoS ONE. (2014) 9:e89454. 10.1371/journal.pone.0089454 24586789 PMC3931790

[61] Biechele G Franzmeier N Blume T Ewers M Luque JM Eckenweber F. Glial activation is moderated by sex in response to amyloidosis but not to tau pathology in mouse models of neurodegenerative diseases. J Neuroinflammation. (2020) 17:374. 10.1186/s12974-020-02046-2 33317543 PMC7737385

[62] Constantinescu CC Mukherjee J. Performance evaluation of an Inveon PET preclinical scanner. Phys Med Biol. (2009) 54:2885–99. 10.1088/0031-9155/54/9/020 19384008 PMC2838767

[63] Luo F Rustay NR Ebert U Hradil VP Cole TB Llano DA. Characterization of 7- and 19-month-old Tg2576 mice using multimodal in vivo imaging: limitations as a translatable model of Alzheimer's disease. Neurobiol Aging. (2012) 33:933–44. 10.1016/j.neurobiolaging.2010.08.005 20961663

[64] Nagy K Tóth M Major P Patay G Egri G Häggkvist J. Performance evaluation of the small-animal nanoScan PET/MRI system. J Nucl Med. (2013) 54:1825–32. 10.2967/jnumed.112.119065 23990683

[65] Mannheim JG Mamach M Reder S Traxl A Mucha N Disselhorst JA. Reproducibility and comparability of preclinical PET imaging data: a multicenter small-animal PET study. J Nucl Med. (2019) 60:1483–91. 10.2967/jnumed.118.221994 30850496

[66] Martorell AJ Paulson AL Suk HJ Abdurrob F Drummond GT Guan W. Multi-sensory gamma stimulation ameliorates Alzheimer's-associated pathology and improves cognition. Cell. (2019) 177:256–71 e22. 10.1016/j.cell.2019.02.014 30879788 PMC6774262

[67] Wang Q Xiao B Cui S Song H Qian Y Dong L. Triptolide treatment reduces Alzheimer's disease (AD)-like pathology through inhibition of BACE1 in a transgenic mouse model of AD. Dis Model Mech. (2014) 7:1385–95. 10.1242/dmm.018218 25481013 PMC4257007

[68] Baranger K van Gijsel-Bonnello M Stephan D Carpentier W Rivera S Khrestchatisky M. Long-term pantethine treatment counteracts pathologic gene dysregulation and decreases Alzheimer's disease pathogenesis in a transgenic mouse model. Neurotherapeutics. (2019) 16:1237–54. 10.1007/s13311-019-00754-z 31267473 PMC6985318

[69] Zeng Y Zhang J Zhu Y Zhang J Shen H Lu J. Tripchlorolide improves cognitive deficits by reducing amyloid beta and upregulating synapse-related proteins in a transgenic model of Alzheimer's disease. J Neurochem. (2015) 133:38–52. 10.1111/jnc.13056 25661995

[70] Meng J Han L Zheng N Xu H Liu Z Zhang X. TMEM59 Haploinsufficiency ameliorates the pathology and cognitive impairment in the 5xFAD mouse model of Alzheimer's disease. Front Cell Dev Biol. (2020) 8:596030. 10.3389/fcell.2020.596030 33195275 PMC7655972

[71] Fiol-deRoque MA Gutierrez-Lanza R Teres S Torres M Barcelo P Rial RV. Cognitive recovery and restoration of cell proliferation in the dentate gyrus in the 5XFAD transgenic mice model of Alzheimer's disease following 2-hydroxy-DHA treatment. Biogerontology. (2013) 14:763–75. 10.1007/s10522-013-9461-4 24114505

